# Dispensing of medicines for asthma and chronic obstructive pulmonary disease through the government health insurance in Syria: a retrospective analysis

**DOI:** 10.1080/16549716.2025.2556526

**Published:** 2025-09-12

**Authors:** Saleh Aljadeeah, Raffaella Ravinetto, Ana Tomas

**Affiliations:** a Department of Public Health, Institute of Tropical Medicine, Antwerp, Belgium; b School of Public Health, University of the Western Cape, Cape Town, South Africa; c Department of Pharmacology, Toxicology and Clinical Pharmacology, Faculty of Medicine, University of Novi Sad, Novi Sad, Serbia

**Keywords:** Quality of Care for Chronic Conditions, Access to medicines, respiratory diseases, primary health care, conflict setting, sanctions

## Abstract

**Background:**

Asthma and chronic obstructive pulmonary disease (COPD) are common noncommunicable diseases, exacerbated in conflict settings by the heightened environmental exposure to triggers, weakened health systems, and poor access to medicines and healthcare. However, accurate data on medicines dispensing in this context are generally scarce.

**Objective:**

We examined the patterns and rates of medicines dispensing for asthma and COPD among the beneficiaries of the Syrian governmental health insurance scheme between June 2018 and March 2021.

**Methods:**

We retrospectively analyzed the outpatient dispensing records for 125,371 adults. Medicines for asthma and COPD were identified using the Anatomical Therapeutic Chemical (ATC) classification system. Dispensing rates were calculated as the number of packages per 1,000 beneficiaries per month, stratified by age, sex, and route of administration.

**Results:**

Out of our sample, 15.02% received at least one package of a medicine for asthma or COPD. Oral formulations were the most frequently dispensed (92.67% of patients), particularly oral salbutamol and xanthines. Inhaled medicines, including inhaled corticosteroids (ICS) and ICS long-acting β2-agonists (ICS–LABA) combinations, were markedly under-dispensed (17.08% of patients). Dispensing rates were higher in females and older adults. Seasonal variation showed peaks in autumn and winter, with a notable decline in April 2020, coinciding with the early COVID-19 period.

**Conclusions:**

The study highlights substantial gaps in dispensing of medicines for asthma and COPD, with particularly low rates for inhalers, likely reflecting barriers driven by the conflict, economic instability, and sanctions. Robust coordinated action is needed to improve their availability in Syria.

## Paper context

- **Main findings**: Oral formulations for asthma and chronic obstructive pulmonary disease were the most frequently dispensed, while inhaled medicines were markedly under-dispensed for the study population.
- **Added knowledge**: This study highlights substantial gaps in dispensing of medicines for asthma and chronic obstructive pulmonary disease likely reflecting barriers driven by the conflict, economic instability, and sanctions.
- **Global health impact for policy and action**: This study contributes to the broader literature on access to medicines for non-communicable diseases in conflict-affected areas and fragile settings, and calls for both short-term and long-term efforts to secure supply and distribution of medicines for these diseases.

## Background

Asthma and chronic obstructive pulmonary disease (COPD) are among the most prevalent noncommunicable diseases (NCDs) and contribute substantially to global morbidity and mortality. Asthma in particular affects an estimated 262 million people worldwide, causing approximately 455,000 deaths annually, and representing a significant health challenge across age groups, particularly in children, where it ranks as the most common NCD [1]. The disease is marked by inflammation and narrowing of the airways, which leads to recurring symptoms such as cough, wheeze, and breathlessness. Symptoms vary in intensity. Severe cases can lead to emergency hospitalizations and, in some instances, death [1]. Prompt administration of reliever medicines such as salbutamol which provide rapid bronchodilation and symptom relief can be lifesaving in severe asthma. Additionally, inhaled corticosteroids play a critical role in preventing severe exacerbations by controlling the underlying airway inflammation [2]. Asthma-related deaths disproportionately occur in low- and middle-income countries (LMICs), where access to diagnostics and medicines are often limited [1].

COPD is a progressive respiratory disease primarily affecting older adults and those with a history of smoking. COPD also increasingly affects populations exposed to indoor air pollution and occupational hazards [3]. It is characterized by persistent limited airflow and chronic respiratory symptoms such as cough, sputum production, and dyspnea, and is one of the leading causes of hospitalization and premature death globally [3]. Acute exacerbations can be life-threatening and frequently require urgent medical interventions. Inhaled bronchodilators, including short-acting beta-agonists and anticholinergics, are essential for the rapid relief of acute symptoms, while long-acting bronchodilators and inhaled corticosteroids play a key role in reducing the frequency and severity of exacerbations [4].

Pharmacotherapy is a cornerstone in the management and control of asthma and COPD and their associated symptoms. Effective control typically relies on a combination of long-term preventive medicines, such as inhaled corticosteroids, and short-acting bronchodilators for acute relief. Both reduce the risk of severe attacks, and improve quality of life [1,5,6]. Conversely, limited access to medicines exacerbates the risk of complications and reduces the likelihood of achieving symptom control [1]. In conflict-affected regions, providing adequate care for asthma and COPD presents significant challenges due to both environmental and health system-related factors [5,7]. First, war increases exposure to pollutants and chemicals which trigger inflammation and narrowing of the airways [5,8]. For instance, during the wars in Afghanistan and Iraq, a significant increase in asthma symptoms and related respiratory issues was observed among soldiers and civil populations. This was reportedly due to high exposure to environmental pollutants, such as smoke from fires and cooking fuel fumes, and to chemical agents used during warfare [8–10]. In Syria, studies have shown a sharp rise in respiratory symptoms, with a significant asthma prevalence in adolescents and adults over-exposed to pollutants and chemical agents. The Global Asthma Network reported an increase in asthma prevalence among Syrian adolescents from 5.2% before the conflict to 19.8% during it, likely due to air pollution, stress, and exposure to irritants [5]. Chemical weapons, including sarin and chlorine gas, have been deployed by the Assad government forces in several attacks in different parts of the country, leading to respiratory health issues, asthma exacerbation, and onset of respiratory diseases [11]. These factors exacerbate respiratory symptoms in those previously diagnosed and trigger asthma onset in vulnerable individuals, especially those in overcrowded shelters where dust, smoke, and other airborne irritants are pervasive [7].

Second, war causes the health system collapse of health systems with disruption of access to diagnosis and medicines, including for asthma and COPD. Essential respiratory medicines such as inhaled corticosteroids are often unavailable, and essential respiratory care is insufficient. Healthcare staff are forced to migrate, or to shift focus to urgent trauma care [5,12]. Humanitarian aid is typically directed toward acute emergencies, with less emphasis on chronic conditions like asthma and COPD [13,14]. The lack of consistent and specialized care means that patients with asthma and COPD experience more complications, uncontrolled symptoms, and decreased quality of life [7,11,12].

The conflict that began in Syria in 2011 led to one of the most severe humanitarian crises of the twenty-first century, causing one of the largest displacement crises in the world, with more than 14 million people forced to leave their homes [15], and resulted in the geographic fragmentation of the country with multiple health systems operating under different political authorities [16]. Until December 2024, the Syrian Ministry of Health managed healthcare in the Assad government-controlled areas. Other administrations oversaw the health system in North Syria: the Autonomous Administration of North and East Syria was in charge in the northeast, while the northwest was divided between opposition-controlled areas, and Turkish-administered regions [16].

To contribute to filling the gap of information on the availability of medicines for asthma and COPD in conflict settings, we retrospectively analyzed the patterns and rates of dispensing of medicines for asthma and COPD in a specific group of adults covered by health insurance within the Assad government-controlled areas of Syria. Through the presentation and analysis of this data, this study provides critical insights into the local availability of medicines for asthma and COPD and establishes important evidence about the impact of conflict on access to essential medicines.

## Methods

### Study setting and data sources

This study was conducted using dispensing data for outpatient medicines. Data was obtained from the governmental health insurance system, which was operational in the areas under the control of the Assad government which controlled the largest part of Syria until December 2024 [17].

The dataset covered a 35-month period from June 2018 to March 2021. For each dispensed medicine, outpatient dispensing records included the product commercial name, dose, dosage form, and administration route. Additional details such as the dispensing date and prescription number were also included. Demographic data of patients was provided, i.e. the age (in years) and sex. We obtained the necessary permission and data use agreement to access and analyse the health insurance dataset used in this study.

### Study population

The study population consisted of 125,371 adults enrolled in the Syrian government health insurance scheme. It included government employees, who primarily represent the country’s middle-income population, as well as members of professional associations and privately insured university students [18]. The government health insurance system covered the costs of outpatient medical services, including prescription medicines for chronic conditions such as asthma and COPD. The dataset was limited to adult individuals actively utilizing the health insurance system for outpatient services within the study’s designated period. The dataset for children and adolescents was not shared and thus is not included in this analysis. In 2019, a total of 841,852 individuals were covered by the government health insurance scheme, of whom approximately 80% were government employees [13,19]. This group included workers from a range of public sectors, such as school teachers, administrative staff, and janitors. In the same year, the total resident population in government-controlled areas was estimated to be around 12 million, suggesting that roughly 7% of the population in these areas was covered by the insurance scheme [20]. This information provides important context for interpreting the representativeness and generalizability of our findings. The national health insurance scheme covers the diagnosis and treatment of selected non-communicable diseases including asthma, COPD, cardiovascular diseases, and diabetes. In contrast, treatment costs for other conditions such as Alzheimer’s, Parkinson’s disease, psychiatric illnesses, and sexually transmitted infections are not covered [13]. In addition to this insurance scheme, healthcare services in Syria are also delivered through public facilities operated by the Ministry of Health, and by private and charitable providers.

## Data analyses

Each medicine was assigned an ATC code based on the ATC/Defined Daily Dose (DDD) classification system (2024) [21]. All medicines classified under the ATC group R03 – *Drugs for Obstructive Airway Diseases* were included. This category encompasses medicines used for the treatment of conditions such as asthma and COPD. The R03-classified medicines were further examined based on ATC subgroups, which distinguish between different types of medicines, such as short-acting (SABA) and long-acting β2-agonists (LABA), inhaled corticosteroids (ICS), leukotriene receptor antagonists, and combinations. The medicines were also categorized by route of administration: inhalation (e.g. metered-dose inhalers, dry powder inhalers, nebulized solutions), oral (e.g. tablets, capsules, syrups), and rectal (e.g. suppositories). The dataset did not include any parenteral formulations. In this study, a ‘package’ is defined as the unit of a medicine as dispensed to patients. This typically corresponds to one commercially available box containing a specific number of dosage units (e.g. tablets, capsules, or inhalers). For inhaled medications such as salbutamol or corticosteroid inhalers, one package is considered as one inhaler device, regardless of the number of doses it contains. This definition aligns with standard practices in drug utilization research, where the unit of analysis often corresponds to the dispensed package, facilitating consistent quantification across different pharmaceutical forms and products [22].

## Statistical analyses

The main outcomes of interest were the number of patients dispensed with any medicines within the R03 category and the number of packages dispensed per 1,000 beneficiaries per month, which provides an estimate of the rate of medicine dispensing in the sample. Age and sex adjusted rates were calculated based on data distribution of the study population. All statistical analyses were conducted using Microsoft Excel, version 2020 [23]. Excel was used for data aggregation, descriptive statistics, and visualization of dispensing over time. IBM SPSS Statistics 22.0 (SPSS Inc., Chicago, IL, USA) software package [24] was used for Chi-square test for categorical variables. Statistical hypotheses were tested at a level of statistical significance (alpha level) of 0.05.

## Ethical considerations

Individual patients cannot be identified through the information in the datasets obtained from the Syrian governmental health insurance as it did not contain any direct identifiers. The study was done in accordance with the principles of Declaration of Helsinki. The study protocol underwent expedited review and was approved by the Institutional Review Board (IRB) and Data Protection Officer of the Institute of Tropical Medicine (Ethical approval number: 1892/25).

## Results

Between June 2018 and March 2021, out of a group of 125,371 insured adults, 39,845 prescriptions of medicines for asthma and COPD (R03 group: obstructive airway diseases) were issued, corresponding to 58,640 packages.

The 39,845 prescriptions were issued to 18,833 patients, representing 15.02% of the overall sample of 125,371. The median age of the patients receiving these prescriptions was 49 years (Interquartile Range: 40–57). Most of them were in the 50–59 age group (38.00%), followed by 40–49 years (36.37%) and 30–39 years (30.41%). Overall, 62.93% of the patients were female and 37.07% were male. The dataset did not include information about the diagnosis, making it impossible to distinguish between patients with asthma and those with COPD.

Out of the 18,833 patients, an overwhelming majority of 17,453 (92.67%) were dispensed oral medicines, while only 3,216 patients were dispensed inhalation products (17.08%). The dispensing rate for inhaled medicines (both inhalers and nebulizing solutions) was of 25.47 packages per 1,000 beneficiaries per month, compared to 39.64 packages per 1,000 beneficiaries per month for oral medicines (Table 1). Minimal dispensing was observed for rectal medicines (<0.1%), only concerning acefylline piperazine (R03DA09).

**Table 1.** Dispensed medicines for asthma and COPD for adult patients with government health insurance in Syria between June 2018 and March 2021.

ATC INN	Number Packages per 1000 person per month	Number of patients (%)
**ORAL**	39.644	17453 (92.67%)
**R03CC – adrenergics for systemic use**	16.860	10780 (57.24%)
R03CC02-salbutamol	0.885	10319 (54.79%)
R03CC03-terbutaline	0.089	563 (2.99%)
R03CC53-terbutaline combinations	15.886	82 (0.44%)
**R03DA – Xanthines**	9.519	6015 (31.94%)
R03DA01 - diprophylline	0.024	3286 (17.45%)
R03DA04-theophylline	1.120	1278 (6.79%)
R03DA05-aminophylline	0.436	894 (4.75%)
R03DA09-acefylline piperazine	2.041	645 (3.42%)
R03DA51-diprophylline combinations	4.662	336 (1.78%)
R03DA54-theophylline combinations	1.235	8 (0.04%)
**R03DC – Leukotriene receptor antagonists**	13.262	3698 (19.64%)
R03DC03-montelukast	12.471	3287 (17.45%)
R03DC53-montelukast combinations	0.791	506 (2.69%)
R03DX – Other systemic drugs for obstructive airway diseases	0.004	2 (0.01%)
R03DX03 -fenspiride	0.004	2 (0.01%)
**RECTAL**	0.006	4 (0.02%)
**R03DA – Xanthines**	0.006	4 (0.02%)
R03DA09-acefylline piperazine	0.006	4 (0.02%)
**INHALATION**	25.470	3216 (17.08%)
**R03AC – selective beta-2-adrenoreceptor agonists**	10.954	2219 (11.78%)
R03AC02-salbutamol	10.953	2218 (11.78%)
R03AC12-salmeterol	0.002	1 (0.01%)
**R03AK – adrenergics in combination with corticosteroids or other drugs, excl. anticholinergics**	11.810	1072 (5.69%)
R03AK06-salmeterol +fluticasone	0.448	846 (4.49%)
R03AK07-budesonide+formoterol	1.144	154 (0.82%)
R03AK11-fluticasone +formoterol	10.179	89 (0.47%)
R03AK13-beclometasone+salbutamol	0.038	16 (0.08%)
**R03AL- adrenergics in combination with anticholinergics incl. triple combinations with corticosteroids**	0.002	1 (0.01%)
R03AL02-salbutamol and ipratropium bromide	0.002	1 (0.01%)
**R03BA – glucocorticoids**	1.545	581 (3.09%)
R03BA01-beclomethasone	0.232	453 (2.41%)
R03BA02-budesonide	1.205	60 (0.32%)
R03BA05-fluticason	0.039	57 (0.30%)
R03BA08-ciclesonide	0.069	19 (0.10%)
**R03BB – anticholinergics**	1.158	200 (1.06%)
R03BB01-Ipratropium bromid	0.685	170 (0.90%)
R03BB04-tiotropium	0.473	35 (0.19%)
**Total**	65.120	18833 (100.00%)

The most frequently dispensed oral medicines were adrenergic agents for systemic use (R03CC), with oral salbutamol (R03CC02) being the most dispensed medicine (54.79% of patients). Xanthines (R03DA, 31.94% of patients), and leukotriene receptor antagonists (R03DC, 19.64% of patients), were also commonly dispensed.

For inhalation products, the most frequently dispensed class was selective beta-2-adrenoreceptor agonists (R03AC, 10.954 packages), particularly salbutamol (R03AC02, 10.953 packages, 11.78% of patients). The dispensing of salmeterol was negligible. Adrenergic-corticosteroid combinations (R03AK) were dispensed to a much smaller number of patients, with highest rates noted for the fluticasone + formoterol combination (10.179 packages per 1,000 beneficiaries per month, 4.49% of patients) (Table 1). Glucocorticoids (R03BA) accounted for 1,545 packages per 1,000 beneficiaries per month 3.09%, with beclomethasone (R03BA01, 2.41% of patients) being the most dispensed.

**Figure 1.**
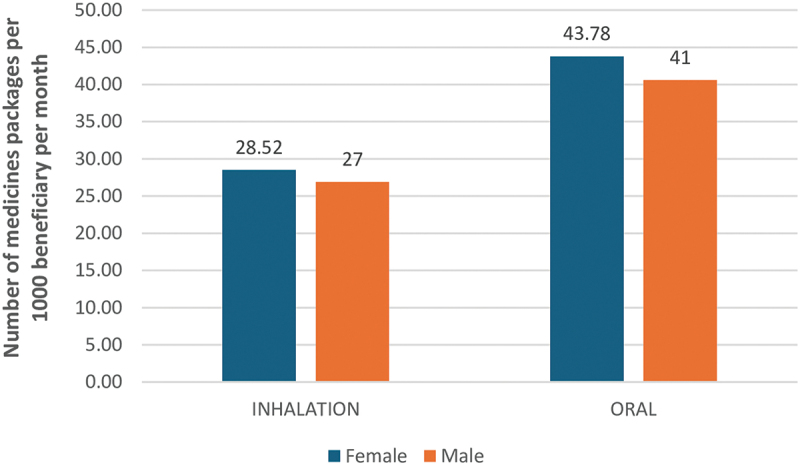
Rates of medicines dispensing by sex and route of administration.

Figure 1 shows the adjusted rate of medicines dispensing (number of packages per 1,000 beneficiaries per month) by sex, stratified by the route of administration (inhalation versus oral). Higher dispensing rates were noted in females, both for inhalation and oral medicines, but there were no statistically significant sex differences in the dispensing rates of different medicine groups (χ^2^ = 3.631, *p* = 0.163).

The highest rates of dispensing were observed for the combination of terbutaline sulfate, bromhexine HCl, guaifenesin and menthol (R03CC53), montelukast (R03DC03), salbutamol (inhalation route, R03AC02) and fluticasone + formoterol (R03AK11) inhalers (Appendix file 1).

**Figure 2.**
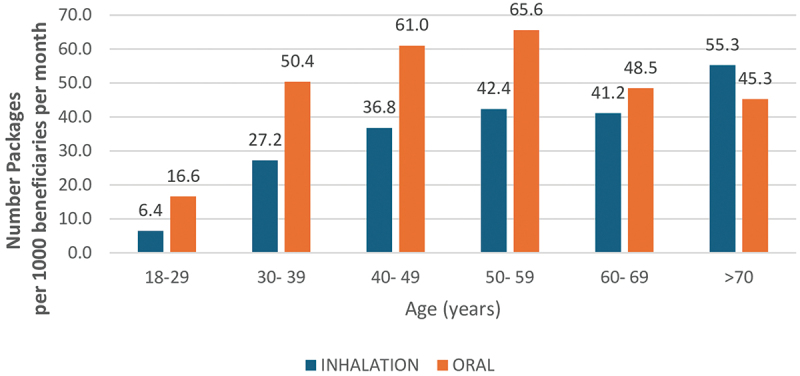
Rates of medicines dispensing by age and route of administration.

There were statistically significant differences between age categories and routes of administration (χ^2^ = 486.689, *p* < 0.001). Inhaled medicines were more commonly dispensed to older patients, surpassing oral medicine only in those aged 70 and above (55.3% vs. 45.3%). The lowest rates of inhaled medicines dispensing were recorded for patients under 30 for both medicine types (Figure 2).

**Figure 3.**
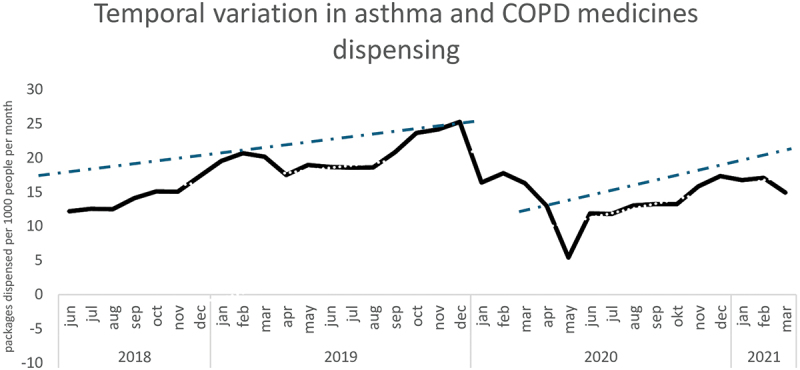
Seasonal variation in medicine dispensing in number of packages per 1000 beneficiaries each month.

The seasonal variation in the rates of medicines dispensing showed fluctuations, with higher rates during autumn and winter and lower rates in summer. There is a noticeable peak in late 2019 and early 2020, followed by a significant drop around April 2020. After mid-2020, the dispensing rate stabilizes with minor fluctuations, gradually rising again toward early 2021 (Figure 3).

## Discussion

To the best of our knowledge, this is the first study to report the patterns and rates of the dispensing of medicines for asthma and COPD in Syria, using health insurance data from a large sample (125,371) of adult beneficiaries, over a 35-month period. This study contributes to the broader literature on access to medicines for NCDs in conflict-affected and fragile settings. While much of the existing literature on asthma and COPD management and medicine use originates from high-income countries, there is less research on these medicines in LMICs, particularly in the Eastern Mediterranean region and in countries affected by conflict [25]. To the best of our knowledge, there are no publicly available datasets or research reports providing national-level figures on the patterns and rates of use of asthma or COPD medicines in Syria. This information gap limits the ability to contextualize the dispensing patterns observed in our study in relation to national consumption. In our study, we observed much higher dispensing rates of oral medicines for asthma and COPD vs inhaled medicines, despite the importance of inhaled products including in the management of acute attacks. The predominance of oral salbutamol and xanthines, coupled with low rates of inhaled corticosteroid (ICS), signals significant gaps in the alignment between clinical best practice, international best practice recommendations and real-world prescribing patterns in the study context [2]. According to the GINA guidelines, ICS in combination with inhaled bronchodilators is the cornerstone of both acute and long-term asthma management. GINA guidelines do not recommend oral salbutamol and theophylline in asthma management. Similarly, the GOLD guidelines for COPD emphasize that inhaled long-acting bronchodilators improve symptoms, lung function, and health status, and reduce rates of exacerbations and are thus central to COPD management. The GOLD guidelines state that theophylline has a small bronchodilator effect in stable COPD, associated with modest symptomatic benefits. The high reliance on oral bronchodilators observed in our study is a substantial deviation from mentioned guidelines, suggesting potential gaps in prescriber adherence or medicine availability and access to inhaled products.

While accurate data on adult asthma and COPD prevalence in Syria is lacking, previous reports have suggested an increase in respiratory symptoms during the conflict, including a rise in adolescent asthma prevalence from 5.2% before the war to 19.8% [26]. In this context, the observed low rates of medicine dispensing, particularly of inhaled therapies, suggest significant challenges in access to medicines. Such challenges are not unique to Syria. Inhalers, such as ICS and ICS–SABA and LABA combinations, play a key role in asthma and COPD control and the prevention of severe or fatal outcomes, with ICS therapy shown to reduce mortality by over 50% and hospitalizations by nearly one-third [26], but remain poorly available or unaffordable in many LMICs. In their absence, oral treatments like theophylline, which are no longer recommended by the Global Initiative for Asthma due to limited effectiveness and higher risk of adverse effects, are still often used [27]. In addition to lack of access, the findings from a multi-country survey conducted among physicians from various LMICs highlighted other common barriers to inhaler use, including patient hesitancy driven by fear of addiction, stigma, or side effects [25]. Other studies confirm a widespread low availability of ICS and combination inhalers. Findings from a 52-country survey, for instance, revealed that ICS inhalers were unavailable in public hospitals in Syria in 2011 [27,28]. Stolbrink and colleagues, who conducted a comprehensive review of availability, cost, and affordability of WHO essential medicines for asthma and COPD in LMICs, reported that ICS inhalers remain of low availability and unaffordable, with prices often exceeding weekly wages for the lowest-paid government workers [29]. Generally, salbutamol remains the most prescribed reliever, and oral therapies are still widely used in the absence of better options. The high reliance on oral bronchodilators observed in our study in Syria is consistent with other LMICs and likely reflects a combination of limited availability and patient-related factors due to interrelated barriers [25].

The economic sanctions imposed on Syria may have compounded other barriers to accessing essential medicines [30]. Studies from Iran illustrated how sanctions targeting banking systems severely limited access to both imported and locally manufactured asthma medicines, as local production often depends on imported raw materials [26,31]. In Tehran, the availability of essential asthma inhalers in retail pharmacies declined from 60% to 28% following the enforcement of banking sanctions in 2013 [26]. These observations are pertinent to the Syrian context, where international sanctions and conflict-related disruptions to supply chains likely played a key role in the limited availability of essential medicines, including for asthma and COPD. In similar settings, shortages have been addressed through informal channels, including smuggling and other practices associated with serious quality concerns [32]. Notably, our study sample represents a relatively privileged group who was still benefiting from an insurance scheme despite the conflict setting. It may be hypothesized that the dispensing rate would be lower if investigating more socially vulnerable groups that pay out-of-pocket for obtaining medicines for asthma and COPD.

In our study, female beneficiaries had slightly higher dispensing rates for both oral and inhaled medicines. These differences may be attributed to greater health-seeking behavior among women, differences in disease burden, or provider bias. Further research is needed to explore these trends. As expected, the rate of asthma medicines dispensing increased with age, with peak dispensing observed in the 50–59 age group. This mirrors trends seen in other NCDs such as diabetes and cardiovascular disease, where medicine use increases with age due to higher disease prevalence [13]. However, our analysis is also influenced by the fact that it is based on a dataset including only adult patients. Trends could be different if children and adolescents were included.

The seasonal variation observed in this study, with higher dispensing rates during autumn and winter and lower rates in summer, aligns with evidence about reactive asthma/COPD and medicines use. Studies in Canada and the U.S. showed that controller and reliever fills tend to peak during periods of known seasonal exacerbations, particularly in autumn and the winter months [33]. However, these patterns also suggest that medicines are often used reactively, in response to symptom worsening, rather than proactively to maintain control at least when asthma and COPD are moderate or severe. The observed fall in dispensing rates during April 2020 may reflect disruptions linked to the early months of the COVID-19 pandemic. Other studies have shown that outpatient visits for asthma initially declined during the pandemic [34].

In Syria, improving access to asthma and COPD essential medicines, especially inhalers, is essential for providing adequate care, within and outside health insurance schemes, for adult and pediatric patients. This requires both immediate and long-term action. In the short term, priority could be given to strengthening supply chain systems, improving coordination among humanitarian actors and the local health systems, and ensuring that essential asthma and COPD medicines, particularly inhaled corticosteroids and ICS–SABA and LABA combinations, are consistently included in procurement and donation programs [27,29]. Local production of inhalers could become an important component of long-term strategies, but this approach faces significant challenges related to the importation of raw materials, excipients and packaging materials for the production of asthma inhalers and, more broadly, to the need for rebuilding the manufacturing capacity in the aftermath of the conflict [26,31]. As Syria moves toward a long-awaited recovery, efforts to strengthen the pharmaceutical system will be critical to ensure equitable access to quality-assured essential medicines, including those for asthma and COPD. The fall of the Assad regime in December 2024 has potentially opened a new chapter for Syria, creating the space and the responsibility to rebuild the health system on more equitable and inclusive foundations. The former regime left behind a legacy of widespread violations of human rights, including the right to health. Moreover, the deeply centralized governance model caused stark inequalities in access to healthcare and essential medicines (Ref). The new government must now take urgent steps to ensure equitable access to essential medicines, including those for chronic diseases such as asthma and COPD. This will require sustained investment in national and subnational capacities, meaningful engagement with health professionals and local actors, and the restoration of trust in public health institutions [30]. Additionally, expanding the reach of government health insurance, or gradually developing a universal health coverage model, should be prioritized to reduce financial hardship and ensure that patients living with NCDs such as asthma and COPD across all regions can access essential medicines. Meanwhile, economic sanctions are still indirectly restricting the import of medicines and medical supplies and continue to undermine access to medicines in the country. Meaningful progress requires sanctions be urgently reconsidered [30].

## Strengths and limitations

This study offers important insights into the patterns and rates of asthma and COPD medicines dispensing in a fragile health system affected by prolonged conflict such as Syria. Its key strength lies in the analysis of a dispensing data for a large sample (125,371 beneficiaries) over a period of nearly three years. The use of standardized ATC classification system allows for international comparisons and contributes to the scarce literature on asthma and COPD care in conflict-affected countries. The dataset included demographic and dispensing information, and enabled stratified analysis by age, sex, and medicine groups. The study also has limitations. Most notably, the available dispensing data did not include the information on the specific clinical diagnoses for which medicines were prescribed, therefore we cannot distinguish between prescriptions for asthma and COPD, nor compare prescribing patterns vs applicable guidelines. Additionally, certain medicines included in our analysis, such as montelukast and theophylline, may occasionally be prescribed for other indications (e.g. allergic rhinitis or other respiratory conditions), and not solely for asthma or COPD. The data only covered individuals enrolled in the government health insurance scheme, which largely includes government employees. Furthermore, the data focused only on adult beneficiaries (older than 18 years). This is a population that does not necessarily represent the broader Syrian public: it is possible that dispensing patterns would change when including children and adolescents in the analysis, and that dispensing rates would be lower in other areas, or in population groups with lower income. Additionally, the number of insured individuals in certain governorates such as Deir ez-Zor, Ar-Raqqa, and Idlib was limited due to limited government control in those areas. Finally, asthma and COPD patients, particularly if not covered by this insurance scheme, may have received their medicines through other mechanisms, such as from humanitarian organizations, other non-governmental sources, or through out-of-pocket purchases. This information is not included in our dataset.

## Conclusion

This study contributes to the broader literature highlighting the challenges in accessing essential medicines for NCDs in conflict-affected and fragile contexts with weakened and fragmented health systems, heightened health risk factors, and limited resources. Our findings highlight the inadequate dispensing of asthma and COPD medicines in a population of insured adults in the Assad government-controlled areas of Syria, with a striking over-reliance on oral therapies and underuse of inhalers. We believe that these patterns reflect barriers to medicine availability exacerbated by the conflict, economic instability, and sanctions. The very low rate of inhalers’ dispensing, despite their central role in asthma and COPD management, is of particular concern, and it could be worse in uninsured communities. Improving access to asthma and COPD medicines in Syria requires coordinated short-term efforts to secure supply and distribution, alongside long-term strategies to strengthen the pharmaceutical system.

## Supplementary Material

STROBE checklist cross sectional.docx

Supplementary file.docx

## Data Availability

The datasets generated and analysed during the current study are not publicly available due to a request from the data provider but are available from the corresponding author on reasonable request.
