# Supplementary material for: Dispensing of medicines for asthma and chronic obstructive pulmonary disease through the government health insurance in Syria: a retrospective analysis
Source: Glob Health Action. 2025 Sep 12;18(1):2556526. doi: 10.1080/16549716.2025.2556526 (PMC12434846; doi:10.1080/16549716.2025.2556526)
Supplement: Supplementary file.docx [file ZGHA_A_2556526_SM8072.docx]

**Supplementary figure 1**: Rates of medicines dispensing by sex
